# Atrial high‑rate episodes and risk of major adverse cardiovascular events in patients with dual chamber permanent pacemakers: a retrospective study

**DOI:** 10.1038/s41598-021-85301-7

**Published:** 2021-03-11

**Authors:** Wei-Da Lu, Ju-Yi Chen

**Affiliations:** Division of Cardiology, Department of Internal Medicine, National Cheng Kung University Hospital, College of Medicine, National Cheng Kung University, 138 Sheng-Li Road, Tainan, 704 Taiwan

**Keywords:** Cardiology, Medical research

## Abstract

Patients with atrial high-rate episodes (AHRE) are at higher risk of major adverse cardiovascular events (MACE). The cutoff threshold for AHRE duration for MACE, with/without history of atrial fibrillation (AF) or myocardial infarction (MI), is unknown. A total of 481 consecutive patients with/without history of AF or MI receiving dual-chamber pacemaker implantation were included. The primary outcome was a composite endpoint of MACE after AHRE ≥ 5 min, ≥ 6 h, and ≥ 24 h. AHRE was defined as > 175 bpm (MEDTRONIC) or > 200 bpm (BIOTRONIK) lasting ≥ 5 min. Cox regression analysis with time-dependent covariates was conducted. Patients’ mean age was 75.3 ± 10.7 years and 188 (39.1%) developed AHRE ≥ 5 min, 115 (23.9%) ≥ 6 h, and 83 (17.3%) ≥ 24 h. During follow-up (median 39.9 ± 29.8 months), 92 MACE occurred (IR 5.749%/year, 95% CI 3.88–5.85). AHRE ≥ 5 min (HR 5.252, 95% CI 2.575–10.715, P < 0.001) and ≥ 6 h (HR 2.548, 95% CI 1.284–5.058, P = 0.007) was independently associated with MACE, but not AHRE ≥ 24 h. Patients with history of MI (IR 17.80%/year) had higher MACE incidence than those without (IR 3.77%/year, p = 0.001). Significant differences were found between MACE patients with/without history of AF in AHRE ≥ 5 min but not AHRE ≥ 6 h or ≥ 24 h. Patients with dual-chamber pacemakers who develop AHRE have increased risk of MACE, particularly after history of AF or MI.

## Introduction

Atrial fibrillation (AF) is a common arrhythmia encountered in clinical practice and is a major cause of preventable thromboembolic disease, namely stroke or systemic embolism^1^. Paroxysmal atrial fibrillation (PAF), which is diagnosed by 12-lead electrocardiography, is transient and infrequent, and may be asymptomatic. The increased use of cardiac implantable electronic devices (CIEDs) has provided the technical ability to monitor atrial rhythm long term, and recent studies have focused on subclinical AF or atrial high-rate episodes (AHRE) detected by CIEDs, even in asymptomatic patients. Results of some studies have demonstrated that AHRE is associated with an increased risk of thromboembolic events^2^. Increased risk of major adverse cardiovascular events (MACE) also have been studied in patients with AF^3^ and occasionally those with AHRE^4^. However, the impact of both history of AF or myocardial infarction (MI) and the duration of AHRE on MACE lacks sufficient evidence to reach a conclusion.

Accordingly, we retrospectively examined the associations between different cutoff durations of AHRE and the incidence rates of MACE in patients with dual chamber permanent pacemakers with or without history of AF or MI.

## Methods

Patients ≥ 18 years of age with dual chamber permanent pacemakers (MEDTRONIC or BIOTRONIK) who were treated in the Cardiology Department of National Cheng Kung University Hospital from January 2015 to August 2019 were recruited. The procedures followed were in accordance with the “Declaration of Helsinki” and the ethical standards of the responsible committee on human experimentation (the Institutional Review Board of National Cheng Kung University Hospital, Tainan, Taiwan (B-ER-108-278)). All included patients provided signed informed consent to participate.

### Data collection and definitions

Patients’ medical history and data of co-morbidities and echocardiographic parameters were collected from chart records for retrospective evaluation. Diabetes mellitus was defined by the presence of symptoms and a casual plasma glucose concentration ≥ 200 mg/dL, fasting plasma glucose concentration ≥ 126 mg/dL, 2-h plasma glucose concentration ≥ 200 mg/dL from a 75-g oral glucose tolerance test, or taking medication for diabetes mellitus, as previously described^5^. Hypertension was defined as in-office systolic blood pressure (SBP) values ≥ 140 mmHg and/or diastolic BP (DBP) values ≥ 90 mmHg or taking antihypertensive medication^6^. Dyslipidemia was defined as low-density lipoprotein ≥ 140 mg/dL, high-density lipoprotein < 40 mg/dL, triglycerides ≥ 150 mg/dL, or taking medication for dyslipidemia^7^. Chronic kidney disease was defined as an estimated glomerular filtration rate (eGFR) < 60 mL/ min / 1.73 m^2^^8^. Acute coronary syndrome was defined as either an acute myocardial infarction (AMI; ST-elevation MI or non-ST elevation MI) or unstable angina^9^. Patients with previous ischemic stroke or transient ischemic attack were considered to have cerebrovascular disease. The history of AF was defined as any documented AF in 12-lead electrocardiography (ECG) or Holter recordings, before the date of pacemaker implantation. AHRE were extracted from the devices via telemetry at each office visit every 3 to 6 months^4^. AHRE electrograms were reviewed by at least one experienced electrophysiologist, who cautiously considered the possibility that AHRE included lead noise, far-field R-waves, or other supraventricular tachy-arrhythmias and visually verified AF in the detected AHRE. Atrial sensitivity was initially programmed to 0.2 mV with bipolar sensing of BIOTRONIK and 0.3 mV with bipolar sensing of MEDTRONIC.

The primary endpoint for this study was the occurrence of MACE as recorded in patients’ charts after the date of implantation of pacemakers, including ST elevation myocardial infarction (STEMI), non-ST elevation myocardial infarction (NSTEMI), unstable angina, heart failure with acute exacerbation^4^, cardiovascular hospitalization (peripheral artery disease or stable angina) and cardiac death. AHRE was defined as atrial rate > 175 bpm (MEDTRONIC) or > 200 bpm (BIOTRONIK) and lasting for at least 5 min of atrial tachyarrhythmia recorded by the devices on any day during the study period. We also divided the different AHRE durations by time, including ≥ 5 min, ≥ 6 h and ≥ 24 h, to evaluate the cutoff threshold for MACE. If the patient had multiple AHREs, the longest AHRE duration was used for analysis. Then, if the patient’s longest AHRE duration was 24 h, this patient would be counted in AHRE ≥ 5 min, ≥ 6 h, and ≥ 24 h.

### Statistical analysis

Among baseline characteristics, categorical variables are presented as percentages. Continuous variables are presented as means and standard deviations if normally distributed and median, interquartile range (IQR) if not normally distributed. Chi-square test or Fisher’s exact test was used for categorical variables, and a 2-sample student’s t test for normally distributed continuous variables or Mann–Whitney U test if not normally distributed. The receiver-operating characteristic (ROC) area under the curve (AUC) of AHRE and the associated 95% confidence intervals (CI) were investigated for associations with future MACE. The cutoff values were chosen based on the results of ROC curve analysis and used to evaluate the associated values of AHRE, in minutes, for determining endpoints. Cox regression analysis was used to identify variables associated with AHRE occurrence, reported as hazard ratios with 95% confidence intervals (CI). Indicators of AHRE ≥ 5 min, ≥ 6 h, and ≥ 24 h were determined separately as time-dependent covariates in multivariable Cox proportional hazards regression and survival curves were generated for patients without MACE. If the p value in univariable analysis was < 0.05, the parameter was entered into multivariable analysis, except for devices, which were essential confounders because of different detecting rates in AHRE definitions. Because LVEF was significantly associated with heart failure (Tables 3 and 4), heart failure was selected for inclusion into multivariable analysis. Because mitral E/e’ ratio was significantly associated with LA diameter, LA diameter was selected for inclusion into multivariable analysis. Because drug history was significantly associated with history of heart failure and myocardial infarction, it was not entered into multivariable analysis. Only mitral E/e’ ratio of echocardiographic parameter was included in multivariable analysis (Table 5). For all comparisons, p < 0.05 was considered statistically significant. All data were analyzed using SPSS statistical package version 23.0 (SPSS Inc. Chicago, IL, USA).

### Ethics statement

The study protocol has been approved by the Institutional Review Board of National Cheng Kung University Hospital. (B-ER-108-278).

### Ethics approval and consent to participate

This study was approved by the ethics committee of National Cheng Kung University Hospital and was conducted according to the guidelines of the International Conference on Harmonization for Good Clinical Practice. All patients provided written informed consent before enrollment.

### Consent for publication

All patients provided signed informed consent before enrollment.

## Results

Between January 1, 2014 and August 31, 2019, a total of 498 patients receiving dual chamber permanent pacemaker at our hospital were initially recruited. Seventeen patients were excluded due to loss of follow-up, inadequate or missing data and not providing informed consent. Therefore, the data of 481 patients were finally included as the analytic sample for this retrospective study.

The mean follow-up period was 39.9 ± 29.8 months after the implantation of dual chamber permanent pacemakers. Table 1 shows baseline demographic and clinical characteristics of all patients based on the occurrence of AHRE ≥ 5 min, ≥ 6 h or ≥ 24 h. Mean age was 75.3 ± 10.7 years and 46.2% were women. The most common indication for dual chamber permanent pacemaker implantation (Table 1) was sick sinus syndrome (70.7%), followed by atrioventricular block (28.1%). High percentages of hypertension (93.8%) and hyperlipidemia (91.9%) suggested a relatively high risk of MACE for the entire study cohort. During follow-up, 188 patients developed AHRE ≥ 5 min, 115 patients developed AHRE ≥ 6 h, and 83 patients developed AHRE ≥ 24 h. Patients with AHRE had significantly lower left ventricular ejection fraction, larger left atrial (LA) diameters and history of documented AF. Components, time to MACE, incidence rates and distribution of MACE are reported in Table 2. The whole follow-up duration represented 1600.25 patient-years of observation, and the total number of MACE was 92 (IR 5.75%/year, 95% CI 3.88–5.85). The proportion of MACE for each separate AHRE duration decreased as AHRE duration increased. Patients with a history of MI at baseline (17.80%/year 95% CI 10.23–22.11) had a higher incidence of MACE than those without previous MI (IR 3.77%/year 95% CI 3.01–4.22; p = 0.001).

**Table 1:** Baseline characteristics of the overall study group.

Variables	All patients (n = 481)	AHREs ≥ 5 min			P	AHREs ≥ 6 h		P	AHREs ≥ 24 h		P
		Yes (N = 188)	No (N = 293)			Yes (N = 115)	No (N = 366)		Yes (N = 83)	No (N = 398)	
Age (years)	77.0,14.0	76.0,15.0	77.0,15.0		0.318	76.0,14.0	77.0,15.0	0.376	77.0,14.0	76.0,15.3	0.880
**Gender**					0.204			0.129			0.199
Male	259(53.8%)	108(57.4%)	151(51.5%)			69(60.0%)	190(51.9%)		50(60.2%)	209(52.5%)	
Female	222(46.2%)	80(42.6%)	142(48.5%)			46(40.0%)	176(48.1%)		33(39.8%)	189(47.5%)	
BMI (kg/m^2^)	24.5,2.9	24.3,3.3	24.6,2.6		0.132	24.1,3.1	24.6,2.7	0.083	24.1,3.7	24.6,2.8	0.077
**Device**					< 0.001			< 0.001			< 0.001
Metronic	320(66.5%)	157(83.5%)	163(55.6%)			102(88.7%)	218(59.6%)		75(90.4%)	245(61.6%)	
BIOTRONIK	161(33.5%)	31(16.5%)	130(44.4%)			13(11.3%)	148(40.4%)		8(9.6%)	153(38.4%)	
**Primary indication**					0.335			0.165			0.045
Sinus node dysfunction	340(70.7%)	139(73.9%)	201(68.6%)			85(73.9%)	255(69.7%)		62(74.7%)	278(69.8%)	
Atrioventricular block	135(28.1%)	46(24.5%)	89(30.4%)			27(23.5%)	108(29.5%)		18(21.7%)	117(29.4%)	
Other	6(1.2%)	3(1.6%)	3(1.0%)			3(2.6%)	3(0.8%)		3(3.6%)	3(0.8%)	
CHA_2_DS_2_-VASc score	3.3 ± 1.3	3.4 ± 1.3	3.2 ± 1.3		0.109	3.5 ± 1.4	3.3 ± 1.3	0.194	3.5 ± 1.4	3.3 ± 1.3	0.191
HAS-BLED	2.3 ± 1.1	2.4 ± 1.1	2.2 ± 1.2		0.069	2.5 ± 1.1	2.3 ± 1.1	0.08	2.4 ± 1.1	2.3 ± 1.1	0.370
Hypertension	451(93.8%)	182(96.8%)	269(91.8%)		0.027	110(95.7%)	341(93.2%)	0.337	79(95.2%)	372(93.5%)	0.557
Diabetes mellitus	250(52%)	99(52.7%)	151(51.5%)		0.810	61(53.0%)	189(51.6%)	0.793	44(53.0%)	206(51.8%)	0.835
Hyperlipidemia	442(91.9%)	181(96.3%)	261(89.1%)		0.005	110(95.7%)	332(90.7%)	0.09	79(95.2%)	363(91.2%)	0.228
History of stroke	28(5.8%)	14(7.4%)	14(4.8%)		0.223	5(4.3%)	23(6.3)	0.439	5(6.0%)	23(5.8%)	0.931
History of myocardial infarction	100(20.8%)	38(20.2%)	62(21.2%)		0.803	24(20.9%)	76(20.8%)	0.981	19(22.9%)	81(20.4%)	0.604
**Heart failure**					0.003			0.011			0.013
Preserved EF	50(10.4%)	29(15.4%)	21(7.2%)			19(16.5%)	31(8.5%)		16(19.3%)	34(8.5%)	
Reduced EF	50(10.4%)	24(12.8%)	26(8.9%)			16(13.9%)	34(9.3%)		9(10.8%)	41(10.3%)	
Chronic liver disease	22(4.6%)	8(4.3%)	14(4.8%)		0.789	7(6.1%)	15(4.1%)	0.373	5(6.0%)	17(4.3%)	0.487
Chronic kidney disease	182(37.8%)	79(42.0%)	103(35.2%)		0.130	57(49.6%)	125(34.2%)	0.003	40(48.2%)	142(35.7%)	0.032
Previously documented Af	126(26.2%)	81(43.1%)	45(15.4%)		< 0.001	60(52.2%)	66(18.0%)	< 0.001	46(55.4%)	80(20.1%)	< 0.001
**Echo parameters**											
LVEF (%)	69.0,13.0	67.0,15.0	70.0,13.0		0.002	66.0,14.0	70.0,13.0	< 0.001	66.0,12.0	70.0,12.6	0.008
Mitral E/e’ ratio	11.1,5.0	11.6,5.0	11.0,5.1		0.474	12.0,6.0	11.0,5.0	0.189	12.0,5.0	11.0,5.0	0.174
LA diameter (cm)	3.8,0.7	3.9,0.6	3.7,0.8		0.002	3.9,0.8	3.7,0.7	0.002	3.9,0.8	3.7,0.7	0.005
RV systolic function (s’, m/s)	12.0,2.0	12.0,2.0	12.0,2.0		0.356	12.0,2.0	12.0,2.0	0.523	12.0,2.0	12.0,2.0	0.393
**Drug prescribed at baseline**											
Antiplatelets	153(31.8%)	58(30.9%)	95(32.4%)		0.718	37(32.2%)	116(31.7%)	0.923	24(28.9%)	129(32.4%)	0.534
Anticoagulants	122(25.4%)	81(43.1%)	41(14.0%)		< 0.001	53(46.1%)	69(18.9%)	< 0.001	42(50.6%)	80(20.1%)	< 0.001
Beta blockers	155(32.2%)	81(43.1%)	74(25.3%)		< 0.001	57(49.6%)	98(26.8%)	< 0.001	44(53.0%)	111(27.9%)	< 0.001
Amiodarone	100(20.8%)	60(31.9%)	40(13.7%)		< 0.001	42(36.5%)	58(15.8%)	< 0.001	33(39.8%)	67(17.8%)	< 0.001
Dronedarone	18(3.7%)	14(7.4%)	4(1.4%)		0.001	12(10.4%)	6(1.6%)	< 0.001	8(9.6%)	10(2.5%)	0.002
Flecainide	2(0.4%)	2(1.1%)	0(0%)		0.152	2(1.7%)	0(0%)	0.057	2(2.4%)	0(0%)	0.029
Propafenone	24(5%)	12(6.4%)	12(4.1%)		0.261	6(5.2%)	18(4.9%)	0.898	5(6.0%)	19(4.8%)	0.634
Sotalol	2(0.4%)	2(1.1%)	0(0%)		0.152	2(1.7)	0(0%)	0.057	1(1.2%)	1(0.3%)	0.316
Digoxin	5(1%)	2(1.1%)	3(1.0%)		1.000	0(0%)	5(1.4%)	0.597	0(0%)	5(1.3%)	0.593
Non-DHP CCBs	19(4%)	11(5.9%)	8(2.7%)		0.086	4(3.5%)	15(4.1%)	1.000	4(4.8%)	15(3.8%)	0.755
RAAS inhibitors	194(40.4%)	74(39.4%)	120(41.1%)		0.705	41(35.7%)	153(41.9%)	0.232	28(33.7%)	166(41.8%)	0.179
Diuretics	70(14.6%)	26(13.8%)	44(15.0%)		0.791	20(17.4%)	50(13.7%)	0.322	16(19.3%)	54(13.6%)	0.180
Statins	166(34.5%)	62(33.0%)	104(35.5%)		0.571	37(32.2%)	129(35.2%)	0.546	28(33.7%)	138(34.7%)	0.870
Metformin	79(16.4%)	24(12.8%)	55(18.8%)		0.083	12(10.4%)	67(18.3%)	0.047	9(10.8%)	70(17.6%)	0.131
SGLT2 inhibitors	5(1%)	2(1.1%)	3(1.0%)		1.000	1(0.9%)	4(1.1%)	1.000	1(1.2%)	4(1.0%)	1.000
Follow-up duration	39.9 ± 29.8	40.8 ± 29.7	39.3 ± 30.0		0.588	39.1 ± 28.2	40.2 ± 30.3	0.736	36.6 ± 25.7	40.6 ± 30.6	0.262
Follow-up times	5.6 ± 4.2	5.8 ± 4.5	5.5 ± 4.1		0.519	5.7 ± 4.4	5.6 ± 4.2	0.917	5.1 ± 3.3	5.7 ± 4.4	0.239

Data are presented as mean ± SD or median, IQR or n (%).

*AF* atrial fibrillation, *AHRE* atrial high-rate episodes, *BMI* body mass index, *EF* ejection fraction, *IQR* interquartile range, *LA* left atrium, *LVEF* left ventricular ejection fraction, *RV* right ventricle, *non-DHP CCBs* non-dihydropyridine calcium channel blockers, *RAAS* renin–angiotensin–aldosterone system, *SGLT2* sodium glucose co-transporters 2.

**Table 2:** Type and incidence of MACEs in the whole cohort.

Types of MACEs	Number	Incidence rate (100 patient-years)	CI 95%	Time to event (months)	Age (years)	Gender (female)	History of Af	History of MI	AHREs > 5mins	AHREs > 6mins	AHREs > 6hrs	AHREs > 12hrs	AHREs > 24hrs
STEMI	2	0.125	(0.02–0.34)	30.5 ± 24.7 (13–48)	66.5 ± 2.1	0(0%)	2(100%)	1(50%)	2(100%)	2(100%)	2(100%)	2(100%)	2(100%)
NSTEMI	23	1.437	(0.78–1.77)	29.9 ± 26.8 (2–99)	78.7 ± 9.2	11(47.8%)	8(34.5%)	15(65.2%)	16(69.6%)	16(69.6%)	9(39.1%)	7(30.4%)	7(30.4%)
Unstable angina	35	2.187	(1.29–2.51)	26.8 ± 24.4 (2–106)	76.2 ± 8.0	11(31.4%)	9(25.7%)	19(54.3%)	23(65.7%)	23(65.7%)	13(37.1%)	11(31.4%)	6(17.1%)
Deteriorated heart failure	23	1.437	(0.78–1.77)	23.4 ± 17.5 (2–82)	75.7 ± 8.5	6(26.1%)	9(39.1%)	14(60.9%)	15(65.2%)	15(65.2%)	10(43.5%)	9(39.1%)	8(34.8%)
Cardiovascular hospitalization	6	0.375	(0.13–0.65)	25.8 ± 24.2 (2–78)	73.8 ± 12.7	3(37.5%)	3(50%)	5(62.5%)	4(66.7%)	4(66.7%)	3(50%)	3(37.5%)	2(33.3%)
Cardiac death	3	0.187	(0.04–0.43)	25.7 ± 19.2 (25–27)	76.5 ± 9.2	0(0%)	3(100%)	2(66.7%)	2(66.7%)	2(66.7%)	1(33.3%)	1(33.3%)	0(0%)
Total event	92	5.749	(3.88–5.85)										

Data are presented as mean ± SD or n (%).

*Af* atrial fibrillation, *AHREs* atrial high-rate episodes, *CI* confidence intervals, *MACEs* major adverse cardiac events, *MI* myocardial infarction, *NSTEMI* non ST-elevation myocardial infarction, *STEMI* ST-elevation myocardial infarction.

### ROC-AUC determination of AHRE cutoff values associated with future MACE

**Figure 1:**
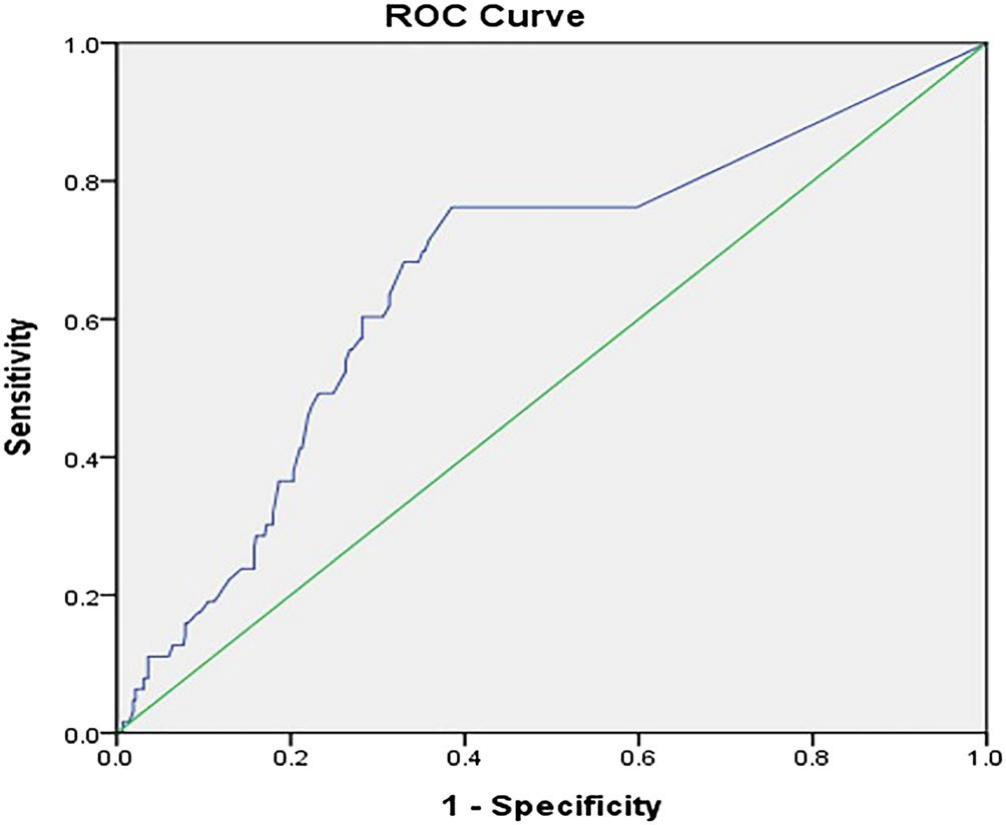
Receiver-operating characteristic curve analysis of atrial high-rate episodes (minutes) in patients with dual chamber permanent pacemakers with subsequent MACE Atrial high rate episodes (minutes): cutoff value, 5-min; sensitivity, 68.3%; specificity, 65.3%; AUC, 0.662; 95% CI, 0.588–0.736; p < 0.001.

The optimal AHRE cutoff value for association with future MACE was determined to be 5-min (sensitivity, 68.3%; specificity, 65.3%; AUC, 0.662; 95% CI, 0.588–0.736; p < 0.001) (Fig. 1).

### Univariable and multivariable Cox regression analysis of associations between duration of AHRE and MACE in all patients

Univariable analysis revealed that the CHA2DS2-VASc score and HAS-BLED score for stroke risk; diabetes mellitus, hyperlipidemia, history of MI, heart failure, and chronic kidney disease; LV ejection fraction, mitral E/e ratio; LA diameter; RV systolic function, and AHRE duration ≥ 5 min, ≥ 6 h and ≥ 24 h; were significantly associated with MACE occurrence in all patients (Table 3). Multivariable Cox regression analysis demonstrated that AHRE ≥ 5 min (HR 5.252, 95% CI 2.575–10.715, p < 0.001) in model A, and AHRE ≥ 6 h (HR 2.548, 95% CI 1.284–5.058, p = 0.007) in model B were independently associated with MACE. However, AHRE ≥ 24 h in model C was not significantly associated with MACE.

**Table 3:** Cox proportional hazard regression analysis with time-dependent covariates for MACE predictors in patients with AHREs ≥ 5 min (Model A),  ≥ 6 h (Model B), ≥ 24 h (Model C).

Variables	All patients (n = 481)	Major adverse cardiac events (MACE)		P	Multivariable Cox regression								
					Model A			Model B			Model C		
		Yes (N = 63)	No (N = 418)		HR	95% CI	*p*	HR	95% CI	*p*	HR	95% CI	*p*
Age (years)	77.0,14.0	77.0,12.0	76.5,16.0	0.467									
**Gender**				0.269									
Male	259(53.8%)	38(60.3%)	221(52.9%)										
Female	222(46.2%)	25(39.7%)	197(47.1%)										
BMI (kg/m^2^)	24.5,2.9	25.1,2.6	24.5,3.0	0.258									
**Device**				0.584	1.252	0.602–2.604	0.548	1.003	0.497–2.022	0.994	0.888	0.447–1.763	0.733
Metronic	320(66.5%)	40(63.5%)	280(67.0%)										
BIOTRONIK	161(33.5%)	23(36.5%)	138(33.0%)										
**Primary indication**				0.212									
Sinus node dysfunction	340(70.7%)	50(79.4%)	290(69.4%)										
Atrioventricular block	135(28.1%)	13(20.6%)	122(29.2%)										
Other	6(1.2%)	0(0%)	6(1.4%)										
CHA_2_DS_2_-VASc score	3.3 ± 1.3	4.3 ± 1.0	3.2 ± 1.3	< 0.001									
HAS-BLED	2.3 ± 1.1	3.3 ± 0.8	2.2 ± 1.1	< 0.001									
Hypertension	451(93.8%)	62(98.4%)	389(93.1%)	0.157									
Diabetes mellitus	250(52%)	52(82.5%)	198(47.4%)	< 0.001	2.536	1.163–5.528	0.019	2.486	1.161–5.320	0.019	2.407	1.131–5.124	0.023
Hyperlipidemia	442(91.9%)	63(100%)	379(90.7%)	0.005	1.550	0.001–1.555	0.998	1.680	0.012–1.869	0.998	1.110	0.015–1.015	0.998
History of stroke	28(5.8%)	4(6.3%)	24(5.7%)	0.775									
History of myocardial infarction	100(20.8%)	31(49.2%)	69(16.5%)	< 0.001	2.796	1.384–5.649	0.004	2.312	1.170–4.569	0.016	2.099	1.073–4.103	0.030
**Heart failure**				< 0.001			0.013			0.010			0.007
Preserved EF	50(10.4%)	11(17.5%)	39(9.3%)		1.170	0.458–2.987	0.743	1.457	0.591–3.592	0.414	1.489	0.611–3.631	0.381
Reduced EF	50(10.4%)	24(38.1%)	26(6.2%)		3.656	1.498–8.921	0.004	3.793	1.592–9.041	0.003	3.960	1.676–9.356	0.002
Chronic liver disease	22(4.6%)	1(1.6%)	21(5.0%)	0.337									
Chronic kidney disease	182(37.8%)	40(63.5%)	142(34%)	< 0.001	1.023	0.498–2.101	0.950	0.933	0.459–1.899	0.849	1.003	0.499–2.018	0.992
Previously documented AF	126(26.2%)	19(30.2%)	107(25.6%)	0.443									
**Echo parameters**													
LVEF (%)	69.0,13.0	57.0,30.0	70.0,11.3	< 0.001									
Mitral E/e’ ratio	11.1,5.0	12.0,7.0	11.0,5.0	0.005									
LA diameter (cm)	3.8,0.7	4.0,0.6	3.7,0.7	< 0.001	1.152	0.694–1.912	0.583	1.185	0.718–1.955	0.507	1.310	0.799–2.148	0.285
RV systolic function (s’, m/s)	12.0,2.0	12.0,2.0	12.0,2.0	< 0.001	0.818	0.658–1.018	0.072	0.814	0.662–1.001	0.051	0.814	0.662–1.000	0.050
**Drug prescribed at baseline**													
Antiplatelets	153(31.8%)	46(73.0%)	107(25.6%)	< 0.001									
Anticoagulants	122(25.4%)	19(30.2%)	103(24.6%)	0.348									
Beta blockers	155(32.2%)	41(65.1%)	114(27.3%)	< 0.001									
Amiodarone	100(20.8%)	25(39.7%)	75(17.9%)	< 0.001									
Dronedarone	18(3.7%)	2(3.2%)	16(3.8%)	1.0									
Flecainide	2(0.4%)	0(0%)	2(0.5%)	1.0									
Propafenone	24(5%)	3(4.8%)	21(5.0%)	1.0									
Sotalol	2(0.4%)	1(1.6%)	1(0.2%)	0.245									
Digoxin	5(1%)	4(6.3%)	1(0.2%)	0.001									
Non-DHP CCBs	19(4%)	3(4.8%)	16(3.8%)	0.726									
RAAS inhibitors	194(40.4%)	28(44.4%)	166(39.8%)	0.485									
Diuretics	70(14.6%)	19(30.2%)	51(12.2%)	< 0.001									
Statins	166(34.5%)	28(44.4%)	138(33.0%)	0.075									
Metformin	79(16.4%)	13(20.6%)	66(15.8%)	0.333									
SGLT2 inhibitors	5(1%)	1(1.6%)	4(1.0%)	0.506									
Follow-up duration	39.9 ± 29.8	41.6 ± 26.4	39.7 ± 30.3	0.630									
Follow-up times	5.6 ± 4.2	5.5 ± 3.5	5.6 ± 4.3	0.790									
AHRE duration ≥ 5 min	188(39.1%)	43(68.3%)	145(34.7%)	< 0.001	5.252	2.575–10.715	< 0.001						
AHRE duration ≥ 6 h	115(23.9%)	26(41.3%)	89(21.3%)	0.001				2.548	1.284–5.058	0.007			
AHRE duration ≥ 24 h	83(17.3%)	17(27.0%)	66(15.8%)	0.028							1.825	0.874–3.809	0.109

Data are presented as mean ± SD or median, IQR or n (%).

*AF* atrial fibrillation, *AHRE* atrial high-rate episodes, *BMI* body mass index, *EF* ejection fraction, *IQR* interquartile range, *LA* left atrium, *LVEF* left ventricular ejection fraction, *RV* right ventricle, *non-DHP CCBs* non-dihydropyridine calcium channel blockers, *RAAS* renin–angiotensin–aldosterone system, *SGLT2* sodium glucose co-transporters 2.

### Univariable and multivariable Cox regression analysis of associations between AHRE duration and MACE in patients with or without history of AF

In the subgroup of patients with or without history of atrial fibrillation, multivariate Cox regression analysis showed that AHREs ≥ 5 min were significantly associated with MACEs in patients without history of AF (HR 4.266, 95% CI 1.856–9.805, p = 0.001) as same as heart failure reduced ejection fraction (HR 5.729, 95% CI 1.917–17.301, P = 0.002) (Table 4). For patients with history of AF, only AHREs ≥ 5 min (HR 18.383, 95% CI 2.006–168.428, p = 0.010) has significant difference (Table 5). Both patients demonstrated that AHREs ≥ 6 h and AHREs ≥ 24 h had no significant difference with MACEs.

**Table 4:** Cox proportional hazard regression analysis with time-dependent covariates for MACE predictors in patients without history of atrial fibrillation and with AHREs ≥ 5mins (Model A), ≥ 6hrs (Model B), ≥ 24hrs (Model C).

Variable	**History of atrial fibrillation (−) (N = 355)**											
	Major adverse cardiac events (MACE)		P	Multivariable Cox regression								
	Yes (N = 44)	No (N = 311)		Model A			Model B			Model C		
				HR	95% CI	*p*	HR	95% CI	*p*	HR	95% CI	*p*
Age (years)	77.0,12.3	77.0,16.0	0.468									
**Gender**			0.115									
Male	30(68.2%)	173(55.6%)										
Female	14(31.8%)	138(44.4%)										
BMI (kg/m^2^)	25.4,2.8	24.6,3.3	0.297									
**Device**			0.929	0.998	0.424–2.352	0.996	0.838	0.365–1.920	0.675	0.707	0.311–1.606	0.408
Metronic	27(61.4%)	193(62.1%)										
BIOTRONIK	17(38.6%)	118(37.9%)										
**Primary indication**			0.229									
Sinus node dysfunction	34(77.3%)	200(64.3%)										
Atrioventricular block	10(22.7%)	110(35.4%)										
Other	0(0%)	1(0.3%)										
CHA_2_DS_2_-VASc score	4.3 ± 1.0	3.1 ± 1.3	< 0.001									
HAS-BLED	3.2 ± 0.7	2.1 ± 1.1	< 0.001									
Hypertension	43(97.7%)	285(91.6%)	0.226									
Diabetes mellitus	37(84.1%)	148(47.6%)	< 0.001	2.577	0.968–6.864	0.058	2.400	0.913–6.307	0.076	2.271	0.872–5.917	0.093
Hyperlipidemia	44(100%)	277(89.1%)	0.013			0.998			0.998	1.130	0.001–1.005	0.998
History of stroke	2(4.5%)	12(3.9%)	0.688									
History of myocardial infarction	23(52.3%)	49(15.8%)	< 0.001	2.087	0.852–5.113	0.107	1.805	0.742–4.394	0.193	1.671	0.701–3.984	0.246
**Heart failure**			< 0.001			0.005			0.005			0.004
Preserved EF	6(13.6%)	22(7.1%)		1.208	0.352–4.150	0.764	1.457	0.432–4.908	0.544	1.577	0.482–5.159	0.376
Reduced EF	20(45.5%)	20(6.4%)		5.759	1.917–17.301	0.002	5.646	1.929–16.523	0.002	5.821	1.988–17.040	< 0.001
Chronic liver disease	0(0%)	18(5.8%)	0.145									
Chronic kidney disease	29(65.9%)	104(33.4%)	< 0.001	0.920	0.375–2.261	0.856	0.836	0.335–2.082	0.700	0.975	0.403–2.358	0.975
**Echo parameters**												
LVEF %	55.0,34.0	70.0,13.0	0.001									
Mitral E/e’ ratio	12.0,6.0	11.1,5.0	0.044									
LA diameter (cm)	4.0,0.7	3.6,0.8	< 0.001	1.502	0.765–2.951	0.238	1.557	0.791–3.064	0.200	1.779	0.902–3.507	0.096
RV systolic function (s’, ms)	11.5,2.0	12.0,2.0	< 0.001	0.856	0.661–1.109	0.239	0.840	0.655–1.077	0.169	0.844	0.399–1.082	0.182
**Drug prescribed at baseline**												
Antiplatelets	36(81.8%)	92(29.6%)	< 0.001									
Anticoagulants	5(11.4%)	27(8.7%)	0.561									
Beta blockers	26(59.1%)	70(22.5%)	< 0.001									
Amiodarone	12(27.3%)	32(10.3%)	0.001									
Dronedarone	1(2.3%)	4(1.3%)	0.486									
Flecainide	0(0%)	0(0%)										
Propafenone	1(2.3%)	14(4.5%)	0.705									
Sotalol	1(2.3%)	1(0.3%)	0.233									
Digoxin	4(9.1%)	0(0%)	< 0.001									
Non-DHP CCBs	2(4.5%)	10(3.2%)	0.650									
RAAS inhibitors	22(50.0%)	116(37.4%)	0.109									
Diuretics	16(36.4%)	41(13.2%)	< 0.001									
Statins	21(47.7%)	100(32.2%)	0.041									
Metformin	10(22.7%)	47(15.1%)	0.198									
SGLT2 inhibitors	1(2.3%)	3(1.0%)	0.412									
Follow-up duration	45.2 ± 27.5	41.7 ± 31.7	0.478									
Follow-up times	6.0 ± 3.8	5.8 ± 4.4	0.759									
AHRE duration ≥ 5 min	25(56.8%)	82(26.4%)	< 0.001	4.266	1.856–9.805	0.001						
AHRE duration ≥ 6 h	14(31.8%)	41(13.2%)	0.001				2.459	0.974–6.210	0.057			
AHRE duration ≥ 24 h	8(18.2%)	29(9.3%)	0.072							1.194	0.399–3.574	0.751

Data are presented as mean ± SD or median, IQR or n (%).

*AF* atrial fibrillation, *AHRE* atrial high-rate episodes, *BMI* body mass index, *EF* ejection fraction, *IQR* interquartile range, *LA* left atrium, *LVEF* left ventricular ejection fraction, *RV* right ventricle, *non-DHP CCBs* non-dihydropyridine calcium channel blockers, *RAAS* renin–angiotensin–aldosterone system, *SGLT2* sodium glucose co-transporters 2.

**Table 5:** Cox proportional hazard regression analysis with time-dependent covariates for MACE predictors in patients with history of atrial fibrillation and with AHREs ≥ 5 min (Model A), ≥ 6 h (Model B), ≥ 24 h (Model C).

Variable	History of atrial fibrillation ( +) (N = 126)														
	Major adverse cardiac events (MACE)			Univariate P valve	Multivariate Cox regression										
	Yes (N = 19)	No (N = 107)			Model A			Model B			Model C				
					HR	95% CI	*p*	HR	95% CI	*p*	HR		95% CI		*p*
Age (years)	75.0,11.0	74.0,13.0		0.733											
**Gender**				0.824											
Male	8(42.1%)	48(44.9%)													
Female	11(57.9%)	59(55.1%)													
BMI (kg/m^2^)	24.8,4.0	24.2,2.6		0.542											
**Device**				0.201	2.363	0.510–10.945	0.272	1.578	0.384–6.479	0.526	1.355		0.340–5.405		0.667
Metronic	13(68.4%)	87(81.3%)													
BIOTRONIK	6(31.6%)	20(18.7%)													
**Primary indication**				0.557											
Sinus node dysfunction	16(84.2%)	90(84.1%)													
Atrioventricular block	3(15.8%)	12(11.2%)													
Other	0(0%)	5(4.7%)													
CHA_2_DS_2_-VASc score	4.3 ± 0.9	3.5 ± 1.3		0.015											
HAS-BLED	3.3 ± 0.9	2.4 ± 1.0		< 0.001											
Hypertension	19(100%)	104(97.2%)		1.000											
Diabetes mellitus	15(78.9%)	50(46.7%)		0.012	2.482	0.644–9.568	0.187	2.820	0.794–10.023	0.109	2.642		0.745–9.368		0.132
Hyperlipidemia	19(100%)	102(95.3%)		1.000											
History of stroke	2(10.5%)	12(11.2%)		1.000											
History of myocardial infarction	8(42.1%)	20(18.7%)		0.024	3.635	0.987–13.384	0.052	3.099	0.941–10.207	0.063	3.020		0.917–9.945		0.069
**Heart failure**				0.026			0.955			0.708					0.549
Preserved EF	5(26.3%)	17(15.9%)			0.928	0.197–4.362	0.924	1.080	0.265–4.397	0.914	1.065		0.262–4.323		0.930
Reduced EF	4(21.1%)	6(5.6%)			1.271	0.188–8.579	0.805	2.125	0.351–12.866	0.412	2.712		0.441–16.664		0.281
Chronic liver disease	1(5.3%)	3(2.8%)		0.484											
Chronic kidney disease	11(57.9%)	38(35.5%)		0.065											
**Echo parameters**															
LVEF %	60.0,16.0	70.0,10.0		< 0.001											
Mitral E/e’ ratio	13.0,10.0	10.6,4.7		0.035	1.090	0.940–1.266	0.255	1.069	0.930–1.229	0.348	1.084		0.944–1.245		0.252
LA diameter (cm)	4.0,0.5	3.9,0.7		0.157											
RV systolic function (s’, m/s)	12.0,3.0	12.0,2.0		0.058											
**Drug prescribed at baseline**															
Antiplatelets	10(52.6%)	15(14.0%)		< 0.001											
Anticoagulants	14(73.7%)	76(71.0%)		0.813											
Beta blockers	15(78.9%)	44(41.1%)		0.003											
Amiodarone	13(68.4%)	43(40.2%)		0.022											
Dronedarone	1(5.3%)	12(11.2%)		0.690											
Flecainide	0(0%)	2(1.9%)		1.000											
Propafenone	2(10.5%)	7(6.5%)		0.624											
Sotalol	0(0%)	(0%)													
Digoxin	0(0%)	1(0.9%)		1.000											
Non-DHP CCBs	1(5.3%)	6(5.6%)		1.000											
RAAS inhibitors	6(31.6%)	50(46.7%)		0.221											
Diuretics	3(15.8%)	10(9.3%)		0.414											
Statins	7(36.8%)	38(35.5%)		0.911											
Metformin	3(15.8%)	19(17.8%)		1.000											
SGLT2 inhibitors	0(0%)	1(0.9%)		1.000											
Follow duration	33.4 ± 22.4	33.8 ± 25.1		0.949											
Follow times	4.4 ± 2.5	5.3 ± 4.0		0.327											
AHRE duration** ≥ **5 min	18(94.7%)	63(58.9%)		0.002	18.383	2.006–168.428	0.010								
AHRE duration** ≥ **6 h	12(63.2%)	48(44.9%)		0.141				2.345	0.715–7.696	0.160					
AHRE duration** ≥ **24 h	9(47.4%)	37(34.6%)		0.286							2.129		0.677–6.692		0.196

Data are presented as mean ± SD or median, IQR or n (%).

*AF* atrial fibrillation, *AHRE* atrial high-rate episodes, *BMI* body mass index, *EF* ejection fraction, *IQR* interquartile range, *LA* left atrium, *LVEF* left ventricular ejection fraction, *RV* right ventricle, *non-DHP CCBs* non-dihydropyridine calcium channel blockers, *RAAS* renin–angiotensin–aldosterone system, *SGLT2* sodium glucose co-transporters 2.

### Univariable and multivariable Cox regression analysis of associations between duration of AHRE and MACEs in patients with or without history of MI

Multivariate Cox regression analysis showed that AHRE ≥ 5 min (HR 4.086, 95% CI 1.638–10.192, p = 0.003), AHRE ≥ 6 h (HR 2.756, 95% CI 1.166–6.517, p = 0.021) and AHRE ≥ 24 h (HR 3.348, 95% CI 1.359–8.243, p = 0.009) were all significantly associated with MACE in patients *without* history of MI (Table 6), but only AHRE ≥ 5 min (HR 10.370, 95% CI 2.860–37.595, p < 0.001) were significantly associated with MACE in patients *with* history of MI (Table 7). Other risk factor such as heart failure reduced ejection was also independently associated with MACE in patients with all three AHRE durations *without* history of MI.

**Table 6:** Cox proportional hazard regression analysis with time-dependent covariates for MACE predictors in patients without history of myocardial infarction and with AHREs ≥ 5 min (Model A), ≥ 6 h (Model B), ≥ 24 h (Model C).

Variable	History of myocardial infarction (−) (N = 381)											
	Mace major adverse cardiac events		Univariate P valve	Multivariate Cox regression								
	Yes (N = 32)	No (N = 349)		Model A			Model B			Model C		
				HR	95% CI	*p*	HR	95% CI	*p*	HR	95% CI	*p*
Age (years)	77.0,11.3	76.0,16.0	0.620									
**Gender**			0.236									
Male	20(62.5%)	180(51.6%)										
Female	12(37.5%)	169(48.4%)										
BMI (kg/m^2^)	25.5,2.5	24.5,2.8	0.184									
**Device**			0.199	0.571	0.201–1.624	0.293	0.530	0.190–1.479	0.225	0.473	0.169–1.324	0.154
Metronic	25(78.1%)	234(67.0%)										
BIOTRONIK	7(21.9%)	115(33.0%)										
**Primary indication**			0.145									
Sinus node dysfunction	27(84.4%)	237(67.9%)										
Atrioventricular block	5(15.6%)	107(30.7%)										
Other	0(0%)	5(1.4%)										
CHA_2_DS_2_-VASc score	3.9 ± 1.0	3.0 ± 1.3	< 0.001									
HAS-BLED	3.0 ± 0.8	1.9 ± 1.0	< 0.001									
Hypertension	32(100%)	321(92.0%)	0.151									
Diabetes mellitus	24(75.0%)	146(41.8%)	< 0.001	3.486	1.401–8.672	0.007	3.468	1.402–8.579	0.007	3.451	1.392–8.553	0.007
Hyperlipidemia	32(100%)	310(88.8%)	0.060									
History of stroke	4(12.5%)	19(5.4%)	0.116									
**Heart failure**			< 0.001			0.045			0.020			0.007
Preserved EF	2(6.3%)	24(6.9%)		0.475	0.093–2.418	0.370	0.621	0.125–3.075	0.559	0.576	0.114–2.903	0.504
Reduced EF	10(31.3%)	11(3.2%)		3.475	1.061–11.379	0.040	4.578	1.428–14.684	0.011	5.399	1.692–17.223	0.004
Chronic liver disease	1(3.1%)	16(4.6%)	1.000									
Previously documented Af	11(34.4%)	87(24.9%)	0.242									
Chronic kidney disease	16(50.0%)	100(28.7%)	0.012	1.162	0.484–2.788	0.737	1.060	0.436–2.578	0.898	1.135	0.474–2.715	0.776
**Echo parameters**												
LVEF %	64.0,28.5	70.0,11.0	0.010									
Mitral E/e’ ratio	12.0,6.2	11.0,4.4	0.075									
LA diameter (cm)	4.0,0.7	3.6,0.8	0.002	1.229	0.665–2.271	0.511				1.291	0.702–2.373	0.411
RV systolic function (s’, ms)	12.0,2.0	13.0,2.0	< 0.001	0.695	0.509–0.948	0.022				0.699	0.516–0.947	0.021
**Drug prescribed at baseline**												
Antiplatelets	23(71.9%)	62(17.8%)	< 0.001									
Anticoagulants	12(37.5%)	80(22.9%)	0.065									
Beta blockers	18(56.3%)	80(22.9%)	< 0.001									
Amiodarone	11(34.4%)	55(15.8%)	0.008									
Dronedarone	0(0%)	13(3.7%)	0.613									
Flecainide	0(0%)	1(0.3%)	1.000									
Propafenone	3(9.4%)	21(6.0%)	0.441									
Sotalol	1(3.1%)	0(0%)	0.084									
Digoxin	2(6.3%)	1(0.3%)	0.019									
Non-DHP CCBs	3(9.4%)	14(4.0%)	0.163									
RAAS inhibitors	11(34.4%)	128(36.8%)	0.787									
Diuretics	8(25.0%)	33(9.5%)	0.007									
Statins	10(31.3)	98(28.1%)	0.703									
Metformin	5(15.6%)	55(15.8%)	0.984									
SGLT2 inhibitors	0(0%)	2(0.6%)	1.000									
Follow-up duration	40.2 ± 27.3	41.9 ± 31.4	0.783									
Follow-up times	5.9 ± 3.9	6.0 ± 4.9	0.923									
AHRE duration ≥ 5 min	24(75.0%)	126(36.1%)	< 0.001	4.086	1.638–10.192	0.003						
AHRE duration ≥ 6 h	16(50.0%)	75(21.5%)	< 0.001				2.756	1.166–6.517	0.021			
AHRE duration ≥ 24 h	11(34.4%)	53(15.2%)	0.005							3.348	1.359–8.243	0.009

Data are presented as mean ± SD or median, IQR or n (%).

*AF* atrial fibrillation, *AHRE* atrial high-rate episodes, *BMI* body mass index, *EF* ejection fraction, *IQR* interquartile range, *LA* left atrium, *LVEF* left ventricular ejection fraction, *RV* right ventricle, *non-DHP CCBs* non-dihydropyridine calcium channel blockers, *RAAS* renin–angiotensin–aldosterone system, *SGLT2* sodium glucose co-transporters 2.

**Table 7:** Cox proportional hazard regression analysis with time-dependent covariates for MACE predictors in patients with history of myocardial infarction and with AHREs ≥ 5 min (Model A), ≥ 6 h (Model B), ≥ 24 h (Model C).

Variable	History of myocardial infarction ( +) (N = 100)														
	Mace major adverse cardiac events			Univariate P valve	Multivariate Cox regression										
	Yes (N = 31)		No (N = 69)		Model A			Model B			Model C				
					HR	95% CI	*p*	HR	95% CI	*p*	HR		95% CI		*p*
Age (years)	78.0,14.0		78.0,10.0	0.887											
**Gender**				0.899											
Male	18(58.1%)		41(59.4%)												
Female	13(41.9%)		28(40.6%)												
BMI (kg/m^2^)	24.8,2.6		23.9,3.5	0.425											
**Device**				0.083	4.881	1.346–17.695	0.016	2.357	0.834–6.663	0.106	1.903		0.669–5.413		0.228
Metronic	15(48.4%)		46(66.7%)												
BIOTRONIK	16(51.6%)		23(33.3%)												
**Primary indication**				0.733											
Sinus node dysfunction	23(74.2%)		53(76.8%)												
Atrioventricular block	8(25.8%)		15(21.7%)												
Other	0(0%)		1(1.4%)												
CHA_2_DS_2_-VASc score	4.7 ± 0.7		4.3 ± 1.0	0.029											
HAS-BLED	3.5 ± 0.6		3.3 ± 0.8	0.268											
Hypertension	30(96.8%)		68%(98.6%)	0.526											
Diabetes mellitus	28(90.3%)		52(75.4%)	0.108											
Hyperlipidemia	31(100%)		69(100%)												
History of stroke	0(0%)		5(7.2%)	0.320											
**Heart failure**				0.012			0.033			0.054					0.035
Preserved EF	9(29.0%)		15(21.7%)		2.728	0.756–9.845	0.125	2.832	0.855–9.381	0.088	3.395		1.017–11.336		0.047
Reduced EF	14(45.2%)		15(21.7%)		5.143	1.477–17.901	0.010	3.565	1.192–10.660	0.023	3.833		1.268–11.585		0.017
Chronic liver disease	0(0%)		5(7.2%)	0.320											
Chronic kidney disease	24(77.4%)		42(60.9%)	0.106											
Previously documented Af	8(25.8%)		20(29.0%)	0.743											
**Echo parameters**															
LVEF %	52.0,27.0		65.0,21.0	0.012											
Mitral E/e’ ratio	13.0,10.0		13.0,7.0	0.687											
LA diameter (cm)	4.0,0.4		4.0,0.7	0.599											
RV systolic function (s’, m/s)	12.0,2.0		12.0,3.0	0.055											
**Drug prescribed at baseline**															
Antiplatelets	23(74.2%)		45(65.2%)	0.373											
Anticoagulants	7(22.6%)		23(33.3%)	0.278											
Beta blockers	23(74.2%)		34(49.3%)	0.020											
Amiodarone	14(45.2%)		20(29.0%)	0.114											
Dronedarone	2(6.5%)		3(4.3%)	0.644											
Flecainide	0(0%)		1(1.4%)	1.000											
Propafenone	0(0%)		0(0%)												
Sotalol	0(0%)		1(1.4%)	1.000											
Digoxin	2(6.5%)		0(0%)	0.094											
Non-DHP CCBs	0(0%)		2(2.9%)	1.000											
RAAS inhibitors	17(54.8%)		38(55.1%)	0.983											
Diuretics	11(35.5%)		18(26.1%)	0.338											
Statins	18(58.1%)		40(58.0%)	0.993											
Metformin	8(25.8%)		11(15.9%)	0.245											
SGLT2 inhibitors	1(3.2%)		2(2.9%)	1.000											
Follow duration	43.0 ± 25.9		28.6 ± 21.2	0.004											
Follow times	5.1 ± 3.1		4.0 ± 3.0	0.105											
AHRE duration ≥ 5 min	19(61.3%)		19(27.5%)	0.001	10.370	2.860–37.595	< 0.001								
AHRE duration ≥ 6 h	10(32.3%)		14(20.3%)	0.195				2.146	0.717–6.419	0.172					
AHRE duration ≥ 24 h	6(19.4%)		13(18.8%)	0.952							0.966		0.274–3.405		0.958

Data are presented as mean ± SD or median, IQR or n (%).

*AF* atrial fibrillation, *AHRE* atrial high-rate episodes, *BMI* body mass index, *EF* ejection fraction, *IQR* interquartile range, *LA* left atrium, *LVEF* left ventricular ejection fraction, *RV* right ventricle, *non-DHP CCBs* non-dihydropyridine calcium channel blockers, *RAAS* renin–angiotensin–aldosterone system, *SGLT2* sodium glucose co-transporters 2.

### Freedom from MACE

**Figure 2:**
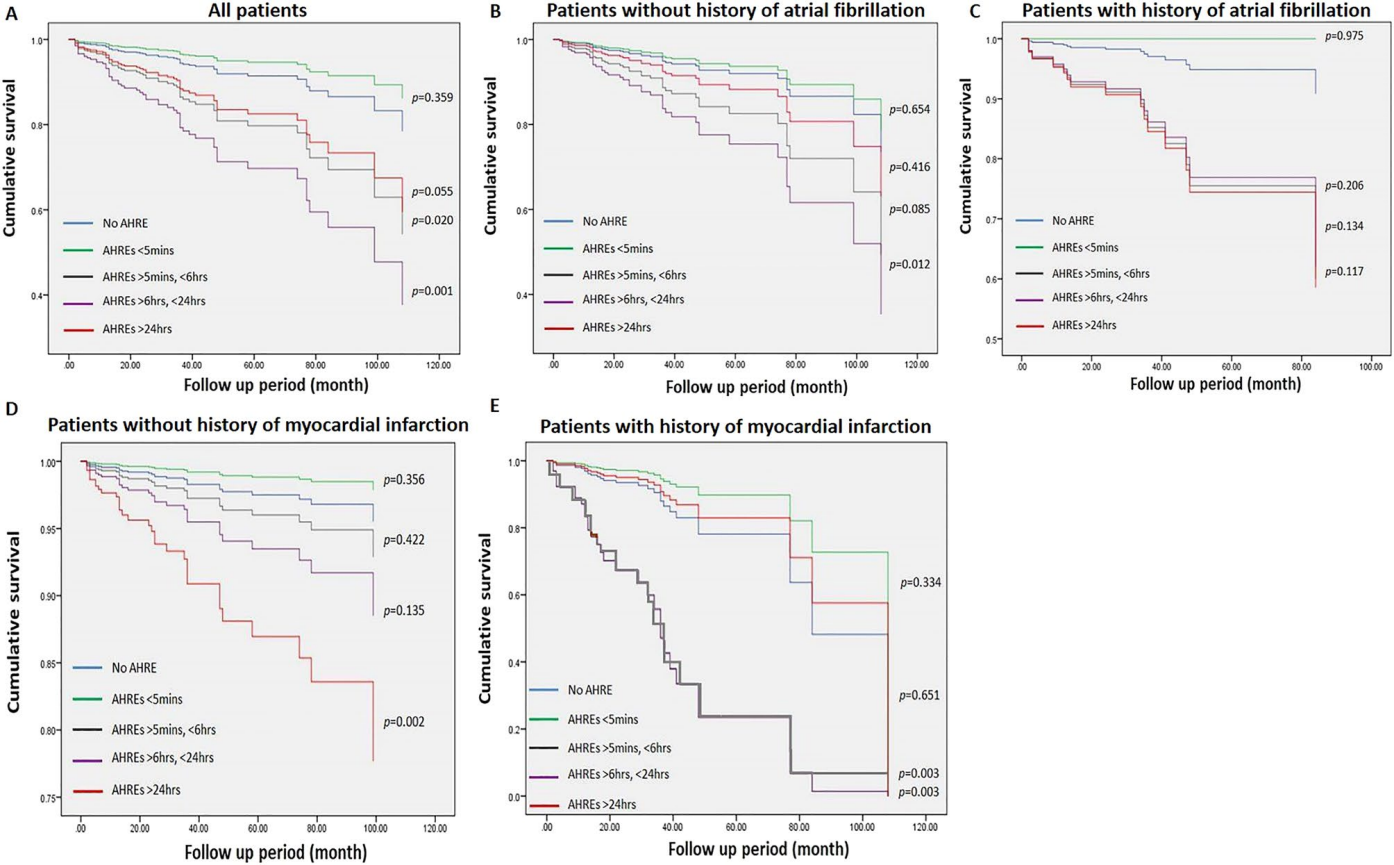
Cox regression event-free survival curves from primary endpoint at 39.9 ± 29.8 months of follow-up based on five subgroups. **(A)** All patients. **(B)** Patients without history of AF. **(C)** Patients with history of AF. **(D)** Patients without history of MI. E: Patients with history of MI. (*AF* atrial fibrillation, *MI* myocardial infarction).

We divided the duration of AHREs into five groups. No AHRE, AHRE < 5 min, AHRE ≥ 5minutes and < 6 h, AHRE ≥ 6 h and < 24 h, and AHRE ≥ 24 h for all patients and with history of AF, history of MI or not. Cox regression survival analysis of all patients showed that only AHRE ≥ 5 min and < 6 h were significantly different compared with patients with no AHRE (Fig. 2). No significant differences were found between patients with no AHRE and any specific duration of AHRE in patients with history of AF. For patients without history of AF, only those with AHRE ≥ 6 h and < 24 h showed significant differences between AHRE duration and MACE occurrence. For patients without history of MI, only AHRE > 24 h had significant differences between AHRE duration and MACE occurrence. In patients with history of MI, AHRE ≥ 5 min and < 6 h, AHRE ≥ 6 h and < 24 h had significant differences between AHRE duration and occurrence of MACE.

## Discussion

The present ‘real world’ cohort study of the associations between different cutoff durations of AHRE and the incidence rates of MACE in patients with dual chamber permanent pacemakers with or without history of AF or MI revealed that (1) almost 40% of patients receiving dual-chamber pacemakers have device-detected AHRE; (2) hypertension, hyperlipidemia, heart failure, history of AF, chronic kidney disease, LA diameter, and AHRE duration are all independent predictors of incident MACE; and (3) although patients with dual chamber pacemakers who develop AHRE are at increased risk of MACE, patients with history of AF or history of MI and the longest AHRE duration also may have higher risk of MACE.

Results of previous studies have demonstrated that AHRE significantly increases risk for MACE^4^ and heart failure^10^, which depends upon the AHRE burden and duration in individual patients. However, in the present study, no linear relationship was found between duration of AHRE and development of MACE. Although AHRE ≥ 5 min and ≥ 6 h were independently associated with MACE, AHRE ≥ 24 h was not. However, in a study with a similar objective, Pastori et al.^4^ found that patients implanted with CIEDs who develop AHRE had a significantly elevated risk of MACE, and that the incidence rate of MACE occurring after AHRE onset was higher in patients with AHRE ≥ 24 h. Although this may correspond with our suggestion that patients with the longest duration of AHRE may be at greater risk of MACE, we did not show this definitively, most likely due to our smaller sample and different definition of MACE.

Results of Pastori et al.^4^ agreed with our results showing that AHRE ≥ 5 min, diabetes and heart failure were independent predictors of MACE. In the present study, we also found that hypertension, hyperlipidemia, history of AF, chronic kidney disease, and increased LA diameter were all significantly associated with the occurrence of AHRE. We also found that patients with MEDTRONIC devices have more frequent occurrence of AHRE than those with BIOTRONIK devices (p < 0.05), which may be due to different default settings for detecting AHRE.

In patients with implantable devices and with no history of AF, device‐detected AHRE can predict long‐term mortality outcomes^11^, and are known to be associated with increased risk of clinical AF, stroke, and thromboembolic events^12^. In the present study, we found that in patients with history of MI, only those with AHRE ≥ 5 min were independently associated with MACE, and for those without history of MI, AHRE ≥ 5 min, ≥ 6 h and ≥ 24 h were all independently associated with MACE. These results suggest that the cutoff value of AHRE may be lower in patients with history of MI than in patients without history of MI, even though the ROC-AUC analysis showed that the optimal cut-off was 5 min.

Three proposed mechanisms of MACE in patients with AF included: (1) both atherosclerosis and inflammatory process yield a pro-thrombotic state; (2) direct coronary thromboembolism from left atrial appendage; and (3) tachycardia episodes resulting in a supply–demand mismatch^13^. However, while AHRE, viewing as subclinical AF, is also recognized as an important clinical entity, therefore it may not always be considered in patients with stroke or transient ischemic attack. As such, AHRE duration remains an important target of research. Future larger prospective studies are needed to explore which duration of AHRE may be the standard cutoff for further evaluation of MACE in patients with AHREs.

Most previous AHRE studies excluded patients with AF history^4,10,14^. We tried evaluating patients with and without AF history in order to identify possible differences. The results showed that only AHRE ≥ 5 min was independently associated with development of MACE, suggesting that in patients with documented history of AF, AHRE may have no important role in the occurrence of MACE.

The other issue we noted was about using anticoagulants in patients with AHRE, even though such a large review of data is not warranted. When we come across a patient with AHRE ≥ 5-min and CHA2DS2-VASc scores > 2 in our daily practice, we follow the current recommendation of 2016 ESC guideline^15^. At the third Joint Consensus Conference of the German Atrial Fibrillation Network (AFNET) and the European Heart Rhythm Association on AF, an algorithm was proposed for management of patients with AHRE^16^. Current updated guidelines recommend that in patients with AHRE ≥ 24 h, clinicians should view them with regard to AF and initiate treatment with a DOAC based on CHA2DS2-VASc scores in order to prevent stroke^16^. Evidence of MACE prevention in AHRE patients is lacking. Results of one study showed that DOAC therapy reduced MI compared with VKA therapy in AF patients^17^. However, other study data showed that the presence of AF was independently associated with a heightened risk of MI despite a lower baseline burden and progression rate of coronary atheroma^18^. Also, aspirin was suggested to have benefit for primary prevention of MACE in specific groups, including among subgroups defined by age, statin use, diabetes and smoking^19^. One study showed that statin use tended to be associated with lower risk of new-onset AF after AMI^20^, but no evidence was found supporting an association between risk and new onset AHRE. Two large ongoing trials (NOAH-AFNET 6 and ARTESiA)^21,22^ will address unmet needs regarding the effectiveness of edoxaban and apixaban for stroke and systemic embolism in patients with AHRE. Further studies are needed to focus on this issue and determine definitively whether patients with new-onset AHRE are at greater risk of MACE, including AF.

Previous studies^23,24^ have shown that AHREs were associated with thromboembolic events in Asian patients. Moreover, two proposed models postulated that atrial cardiomyopathy might play a key role between AHRE and the risk of future ischemic stroke^25,26^. Systemic vascular risk factors accompanied aging can lead to abnormal atrial substrates subsequently resulting in atrial cardiomyopathy, which interacts with hypercoagulability and may be related to atrial dilatation, atrial inflammation/fibrosis, endothelial dysfunction, and/or mechanical dysfunction.

### Limitations

The present study has several limitations. First, this is a single-center, retrospective, and observational study in a hospital-based setting with a relatively small number of included patients, and all patients were Taiwanese. As a result, causality cannot be inferred between AHRE and MACE and results may have been affected by confounding factors. Also, results cannot likely be generalized to other populations. Second, AHRE may have been underestimated due to different default settings for AHRE in devices designed by different companies. The device was viewed as a confounder in the multivariable analysis and was not an independent factor for MACE. Prospective multicenter studies with larger samples are required to confirm results of the present study.

## Conclusion

Patients with dual chamber pacemakers who develop AHRE have significantly increased risk of MACE, particularly those with history of AF or history of MI. However, although this patient population is at increased risk of MACE, the impact on MACE by different cutoff points for AHRE duration in different subpopulations such as those with history of AF or MI must be considered when evaluating risk. Patients with or without history of AF history may have the same cutoff for predicting MACE, but those with MI history may have a lower cutoff point than those without MI.

## Data Availability

All data generated or analysed during this study are included in this published article.
